# Choosing the abdominal incision for the surgical management of severe placenta accreta spectrum: Patient satisfaction and long‐term safety of the Soleymani and Collins transverse abdominal incision

**DOI:** 10.1002/ijgo.16137

**Published:** 2024-12-31

**Authors:** Hooman Soleymani majd, Aakriti Aggarwal, Lamiese Ismail, Annie E. Collins, Prasanna Supramaniam, Lee Lim, Susan Addley, Alicia Hunter, Lexie Pert, Sally L. Collins

**Affiliations:** ^1^ Department of Gynaecology Oncology, Churchill Hospital Oxford University Hospitals NHS Foundation Trust Oxford UK; ^2^ Nuffield Department of Women's and Reproductive Health University of Oxford Oxford UK; ^3^ Department of Gynaecology, John Radcliffe Hospital Oxford University Hospitals NHS Foundation Trust Oxford UK; ^4^ Medical Sciences Division University of Oxford Oxford UK; ^5^ University Hospitals of Derby and Burton NHS Foundation Trust Derby UK; ^6^ Birmingham Women's Hospital Birmingham UK

**Keywords:** abdominal entry, caesarean section, long‐term outcomes, morbidity, novel, placenta accreta

## Abstract

Comparing a novel transverse abdominal entry technique to midline laparotomy for managing severe placenta accreta spectrum, demonstrating excellent long‐term outcome and improved patient satisfaction.

The skin incision from any surgical procedure is a “mark for life,” worn by the patient long after the operation is over. The technique selected mostly depends on the surgeon's experience, preference and the clinical condition of the patient.^1^ Most women with severe placenta accreta spectrum (PAS) are subjected to midline laparotomy due to concerns about surgical access and risk of catastrophic hemorrhage.^2^ We believe that a vertical incision is unnecessarily morbid and disfiguring, resulting in further negative psychological impact for patients already at high risk of post‐traumatic stress disorder (PTSD).^3^

In 2016, the surgical lead for the Oxford Placenta Accreta Team (OxPAT) developed an abdominal entry technique, the Soleymani and Collins (SAC) incision, which amalgamated an extended, transverse, curvilinear incision on the skin and anterior sheath, with a vertical midline incision on the posterior sheath. Rolling the sheath up over the rectus abdominus muscles to the level of the umbilicus, rather than cutting the muscles, facilitating improved wound healing, reducing risk of incisional hernia and producing a more aesthetically pleasing scar. This results in a positive psychological benefit while simultaneously allowing retroperitoneal access, making pelvic devascularization possible and enhancing surgical safety by ureteric identification and bladder mobilization. To our knowledge this is the first abdominal incision described in detail in the literature which uses this approach in an obstetric setting.

This SAC abdominal entry technique was then incorporated into a “25‐step” method for managing severe placenta accreta spectrum, published in 2019.^4^ A detailed description including diagrams, photos, and videos demonstrating the incision technique is available at www.placentaaccretasspectrum.com.

Our original paper^4^ demonstrated non‐inferiority to a midline vertical incision for anatomical access and immediate surgical morbidity, but long‐term outcomes, including patient satisfaction, remained unknown.^4^ To validate this new incision technique, assessment of long‐term patient satisfaction was warranted.

Ethical approval was obtained to undertake this prospective questionnaire study (NHS REC22/SS/105). We attempted to contact the 24 patients included in our original study^4^ and all patients who had a caesarean hysterectomy for severe PAS (International Federation of Gynecology and Obstetrics [FIGO] FIGO grade 3b or c) who had delivered more than 1 year before this study commenced. In total, 27 patients with severe PAS were delivered between January 2011 and December 2022 in our UK tertiary referral unit. Between 2011 and 2016, all patients had midline laparotomy. After 2016, patients typically had the SAC incision, which had replaced midline laparotomy (our previous standard of care).

The patients were contacted by post or email inviting them to participate. Enclosed with the letter or email were a participant information sheet, a consent form, and a questionnaire. Upon return of the signed consent form, a call was arranged to complete the questionnaire on the telephone. In the absence of any response after 2 weeks, a second letter or email was sent, after which no further contact was attempted.

Due to the small sample size, nonparametric testing was used with Fisher's exact test for binary data and the Mann–Whitney test for continuous variables. Significance was set at *P* < 0.05. Analysis was conducted using the Statistical Package for Social Sciences (V26.0; IBM Corporation, USA).

The results are shown in Table 1. Of the 27 patients contacted, 17 initially responded. Four patients from the midline vertical incision group declined to participate after responding (two cited psychological trauma related to childbirth and two initially consented and then declined to complete the questionnaire with no reason given). In total, 13 patients completed the questionnaire. Of these, eight had received the SAC incision and five had midline vertical incision. Due to the change of practice occurring in 2016, the time since surgery between the two groups was significantly different (*P* = 0.004), with the median time from delivery for the vertical incision being 11 years and 2.5 years for the SAC group.

**TABLE 1:** Summary of patient demographics, short‐term maternal morbidity and long‐term patient satisfaction.

	SAC incision	Vertical incision	*P*‐value
Number of patients contacted	11	16	‐
Number of patients initially responded	8	9	‐
Number of patients who declined to participate	0	4	‐

Patient demographics	SAC incision (*n* = 8)	Vertical incision (*n* = 5)	*P*‐value
Age at the time of surgery	33 (5.7)	35 (3.1)	0.24^a^
Body mass index at the time of surgery	26.8 (4.7)	26.2 (1.6)	0.28^a^
Time since surgery (years)	2.5 (2.2)	11 (1.7)	0.004^a^
Gestation at delivery (completed weeks)	35.5 (1.7)	35 (0.8)	1^a^
Maternal morbidity			
Estimated blood loss (mL)	2850 (1494)	2000 (2610)	0.88^a^
Received blood products	2	2	1^b^
Significant transfusion (>5 units red cells)	0	1	1^b^
Massive transfusion (>10 units red cells)	0	1	0.38^b^
ICU admission with organ failure	0	1	0.38^b^
Accidental bladder injury	1	1	1^b^
Ureteric Injury	0	1	0.38^b^
Vesicovaginal fistula	0	1	0.38^b^
Postoperative sepsis^c^	0	0	N/A
Patient reported satisfaction results^a^			

How satisfied are you about the cosmetic appearance of your scar? (1 = extremely dissatisfied, 10 = extremely satisfied)	10	7	0.04
Is the scar painful? (1 = not at all, 10 = yes very much)	1.5	1	0.86
Is the scar itchy? (1 = not at all, 10 = yes very much)	1	2	0.53
Is the color of the scar different from your normal skin? (1 = not at all, 10 = yes very much)	1	3	0.35
Is the stiffness of the scar different from your normal skin? (1 = not at all, 10 = yes very much)	2	8	0.09
Is the thickness of the scar different from your normal skin? (1 = not at all, 10 = yes very much)	4.5	8	0.13
Is the scar more irregular your normal skin? (1 = not at all, 10 = yes very much)	3	8	0.10

Patient reported adverse outcomes^b^			
Altered sensation in groin/inner thigh	4	0	0.105
Burning in lower abdomen	3	0	0.231
Altered sensation in front thigh	2	0	0.487
Inability to open bowels/pass wind	0	0	‐
Bulging tissue under scar tissue	0	1	0.385
Swelling or fluid collection under scar	0	0	‐

Abbreviation: ICU, intensive care unit.

^a^ Mann Whitney *U*‐test with results given as median (standard deviation) and

^b^ Fisher's exact test were used calculated *P*‐values.

^c^ Defined by the Third International Consensus Definitions for Sepsis and Septic Shock (Sepsis‐3).^5^

There was no significant difference between the two follow up groups in terms of age at delivery, body mass index, surgical morbidity, and blood loss. Despite the small sample size, patients who had the SAC incision reported significantly higher satisfaction with cosmetic appearance compared to those who had a vertical incision (*P* = 0.04). While almost all of the other patient scar assessments such as pain, color, and stiffness were better in the SAC incision group, they failed to reach statistical significance. Some patients reported an alteration in skin sensation with the SAC incision, which was not seen with the midline laparotomy, although again this did not reach statistical significance.

Severe PAS (FIGO grade 3b or c) is extremely rare; therefore, the sample size for any study limiting itself to this end of the spectrum is, by its very nature, going to be small. We chose to only include severe PAS in our original study as these are the cases where surgeons are most likely to opt for a vertical midline incision due to the belief that transverse will not provide sufficient surgical access. We followed these women up as we had already proven non‐inferiority of the SAC incision for short‐term surgical morbidity. Childbirth complicated by PAS frequently adversely affects maternal mental health, resulting in anxiety and PTSD.^2^, ^3^ While this might have had a negative effect on the number of patients wishing to participate, our response rate (48%) is close to the reported average scientific questionnaire study response rate of 55.6% (standard deviation 19.7%).^6^ We acknowledge that smaller sample sizes make statistical interpretation difficult. Nonetheless, there was a significantly increased cosmetic satisfaction with the SAC technique and a trend towards less scar concerns in terms of stiffness and color. This finding is consistent with the anecdotal evidence.

This paper demonstrates long‐term patient outcomes that have hitherto been missing from studies on surgical techniques used for complex caesarean section. Based on our previous study,^4^ we contend that our novel technique is non‐inferior to vertical midline laparotomy in terms of anatomical access and maternal morbidity. Therefore, we believe that patients with severe PAS should be offered this incision for their surgery but must be warned about the risk of altered skin sensation when making an informed decision regarding their choice.

## AUTHOR CONTRIBUTIONS

All authors meet the ICMJE criteria for authorship, have substantially contributed to the acquisition, analysis, and data interpretation, and reviewed the paper critically for important intellectual content. Furthermore, they have approved the manuscript’ s final version and agreed to be accountable for the work.

## FUNDING INFORMATION

None.

## CONFLICT OF INTEREST STATEMENT

The authors declare no conflicts of interest.

## Data Availability

The data that support the findings of this study are available from the corresponding author upon reasonable request.
